# Apheresis CD8^+^CCR7^+^CD45RA^−^ T-Cells as a Novel Biomarker Associated with CAR T-Cell Kinetics and Clinical Outcome

**DOI:** 10.3390/ijms27020866

**Published:** 2026-01-15

**Authors:** Iván García de la Torre, Carlota García-Hoz, Fernando Martin-Moro, José Ignacio Fernández-Velasco, Kyra Velázquez-Kennedy, Eulalia Rodríguez-Martín, Alejandro Luna De Abia, Ernesto Roldán, Gemma Moreno Jiménez, Javier López-Jiménez, Luisa María Villar, Roberto Pariente-Rodríguez

**Affiliations:** 1 Department of Immunology, Instituto Ramón y Cajal de Investigación Sanitaria (IRYCIS), Hospital Universitario Ramón y Cajal, 28034 Madrid, Spain; igtorre@salud.madrid.org (I.G.d.l.T.);; 2 Department of Hematology, Instituto Ramón y Cajal de Investigación Sanitaria (IRYCIS), Hospital Universitario Ramón y Cajal, 28034 Madrid, Spain

**Keywords:** CAR-T, diffuse large B-cell lymphoma, flow cytometry, apheresis, central memory T-cells, biomarkers

## Abstract

Chimeric antigen receptor (CAR) T-cell therapy has revolutionized the treatment of relapsed or refractory (r/r) diffuse large B-cell lymphoma (DLBCL); however, a significant proportion of patients fail to achieve a durable response, underscoring the need for reliable predictive biomarkers. We characterize T-lymphocyte subpopulations in apheresis samples from 23 r/r large B-cell lymphoma (LBCL) patients who received axicabtagene ciloleucel (axi-cel) to identify pre-treatment cell biomarkers associated with CAR T-cell kinetics and clinical outcomes. Immunophenotyping of T-cells within fresh apheresis samples and monitoring of circulating CAR T-cells were performed by multiparametric flow cytometry. The median peak CAR T-cell count was 45.2 CAR T-cells/mL. Strong CAR-T expanders (≥45.2 CAR T-cells/mL) exhibited higher values of both CD4^+^ (*p* = 0.011) and CD8^+^ (*p* = 0.023) central memory T-cells (T_CM_; CCR7^+^CD45RA^−^), as well as lower proportions of CD8^+^CD38^+^ T-cells in apheresis samples. In apheresis, a cut-off value of >4.3% of CD8^+^ T_CM_ predicted strong CAR-T expansion (AUC: 0.80; *p* = 0.023) and superior progression-free survival (*p* = 0.04) compared with patients who had CD8^+^ T_CM_ below the cut-off. Our data suggest that high frequencies of CD8^+^ T_CM_ cells in apheresis samples may represent a promising pre-treatment biomarker associated with strong CAR-T expansion and superior clinical outcome in r/r LBCL patients following axi-cel.

## 1. Introduction

Chimeric antigen receptor (CAR) T-cell therapy has revolutionized cancer immunotherapy in recent years. Conventional treatments used in B-cell malignancies do not always achieve complete eradication of malignant cells [1]. Among patients with relapsed/refractory (r/r) diffuse large B-cell lymphoma (DLBCL), the proportion achieving a 1-year overall survival rate is low [2]. Commercial CAR T-cell therapy, which engineers a patient’s T-cell to recognize CD19 antigen on malignant cells, has emerged as a promising treatment for r/r DLBCL. Pivotal trials have reported an overall response rate of 82–52% and a complete response rate of 54–40% in third line [3,4,5]. CAR-T therapy has also shown encouraging efficacy when investigated as a second-line approach for large B-cell lymphomas (LBCLs) [6,7]. In addition, long-term follow-up showed the curative potential of CAR T-cell therapy in refractory LBCL [8,9]. Although these results are very encouraging, a significant proportion of patients still fail to respond or experience relapse. Therefore, identifying biomarkers that can help stratify patients most likely to benefit from CAR T-cell therapy becomes essential.

Clinical data show a positive correlation between magnitude and duration of in vivo CAR T-cell expansion with treatment efficacy [10]. These parameters are valuable for immunomonitoring after treatment infusion, but they are not useful for predicting treatment response prior to administration. Previous studies have also mainly focused on CAR-T products. In this line, less differentiated T-phenotypes, such as naïve and stem-cell memory subsets, in CAR-T products have been associated with therapeutic efficacy [11,12]. Another study showed that enrichment of the CD8+ T central memory phenotype in CAR T-cell products was associated with improved in vivo CAR T-cell expansion and response [13]. However, the role of these potential biomarkers has not been fully studied in the starting material used in CAR T-cell manufacturing: the apheresis product.

In this study, we addressed this issue by performing immunophenotypic characterization of T-cells within apheresis samples and evaluated their association with CAR T-cell kinetics and clinical efficacy in patients with r/r LBCL. Flow cytometry analysis of the apheresis product indicated that central memory T (T_CM_; CCR7^+^CD45RA^−^) cells are associated with robust CAR T expansions. In addition, patients with higher frequencies of apheresis CD8^+^ T_CM_ cells had improved clinical outcomes following axicabtagén ciloleucel (axi-cel). These findings provide new insights into the pre-treatment evaluation of patients eligible for CAR T-cell therapy and may report strategies to optimize manufacturing protocols in order to improve therapeutic efficacy.

## 2. Results

### 2.1. Patients

We included 23 consecutive heavily pre-treated patients with either r/r LBCL who received axi-cel between December 2022 and March 2025 at Ramón y Cajal University Hospital. The cohort comprised 14 (61%) males and 9 (39%) females, with a median age of 65 years (IQR: 54.5–70 years) at the time of infusion. Table 1 summarizes the clinical characteristics of the patients included in the study. Immunophenotyping of the CAR-T therapy starting material was performed by flow cytometry on fresh apheresis. After infusion, commercial CAR T-cell kinetics were monitored by flow cytometry in peripheral blood. Acute adverse events, such as cytokine-release syndrome (CRS) and immune effector cell-associated neurotoxicity syndrome (ICANS), and clinical outcomes were subsequently evaluated.

### 2.2. CAR T-Cell Kinetics

**Figure 1:**
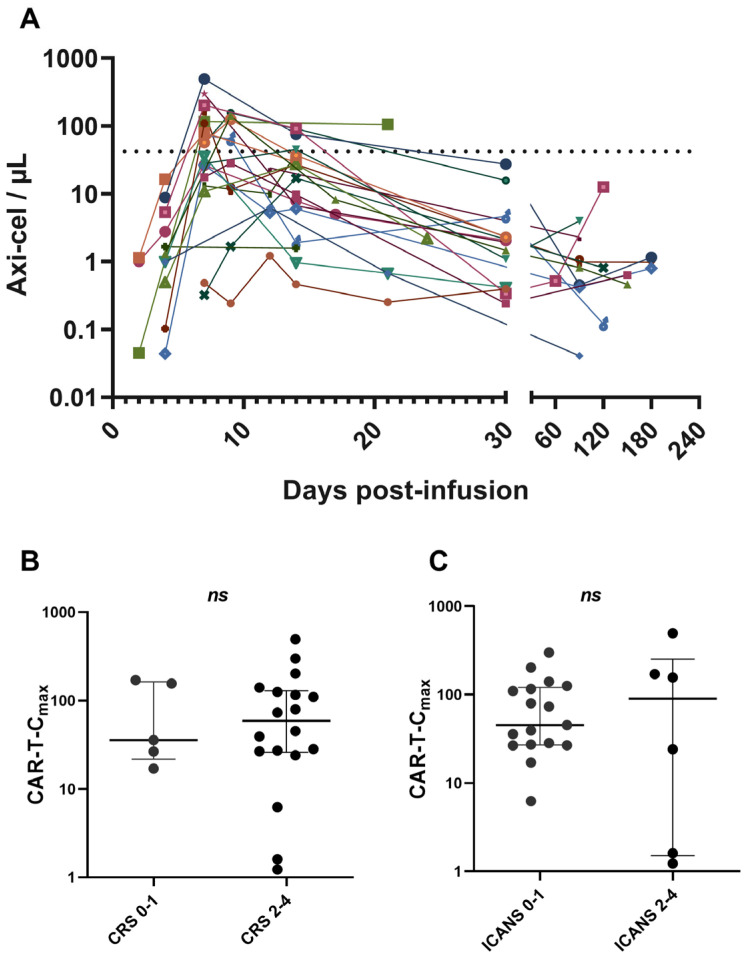
Dynamics of CAR T-cell expansion and association with toxicity. (**A**) Commercial CD19 CAR T-cell kinetics post-infusion determined by flow cytometry in 23 patients with r/r LBCL. Absolute axi-cel counts were determined by dual platform. (**B**) Correlation between CAR-T-C_max_ and severity of CRS. (**C**) Correlation between CAR-T-C_max_ and severity of ICANS. The dashed line indicates the median of CAR-T-C_max_. Data are presented as median with interquartile range. Values ≥ 0.05 were considered not statistically significant (*ns*).

**Figure 2:**
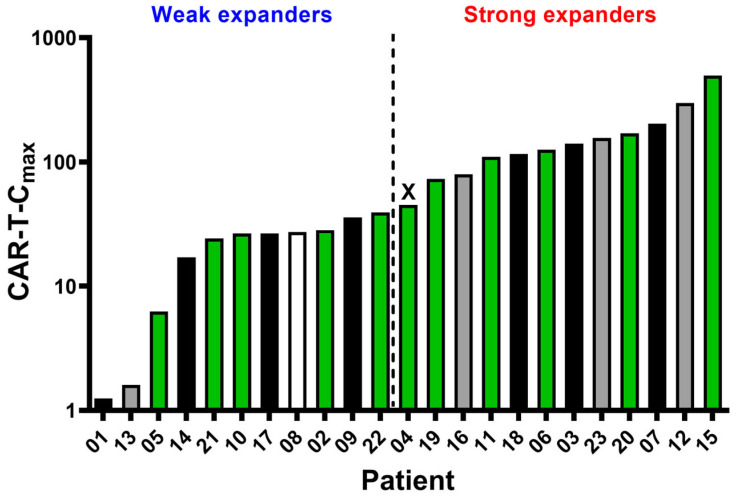
Comparison of axi-cel peak expansion (CAR-T-C_max_) and clinical outcome for individual patients. The median CAR-T-C_max_ value (45.2 CAR T-cells/mL) was found to be patient 4 (represented as X) and was used to set weak and strong CAR-T expanders. Fill pattern represents clinical outcome: black—deceased; grey—progression/relapse disease; green—complete response; and white—no information.

One hundred and twelve samples from 23 patients were analysed to evaluate axi-cel kinetics (Figure 1A). Quantification and characterization of CAR T-cells were performed using multiparametric flow cytometry. Absolute counts of axi-cel, expressed as CAR T-cells per µL of peripheral blood, were determined using a dual-platform method. Globally, anti-CD19 CAR T-cells reached peak expansion in peripheral blood (CAR-T-C_max_) within 14 days after infusion, predominantly at day +7 (Figure 1A). CAR-T-Cmax did not show a correlation with the severity of cytokine release syndrome CRS or ICANS (Figure 1B,C). CAR-T-C_max_ ranged from 1.22 to 494.74 CAR T-cells/µL, with a median of 45.2 CAR T-cells/µL (Figure 2). Patients exhibiting CAR-T-C_max_ values below the median were categorized as weak expanders, whereas those with values at or above the median were classified as strong expanders (Figure 2). At the time of CAR-T-C_max_, no significant differences in the CD4/CD8 ratio of CAR^+^ cells were detected between weak and strong expanders (Figure S4).

### 2.3. Apheresis T-Cell Subsets Impact CAR T-Cell Kinetics

**Figure 3:**
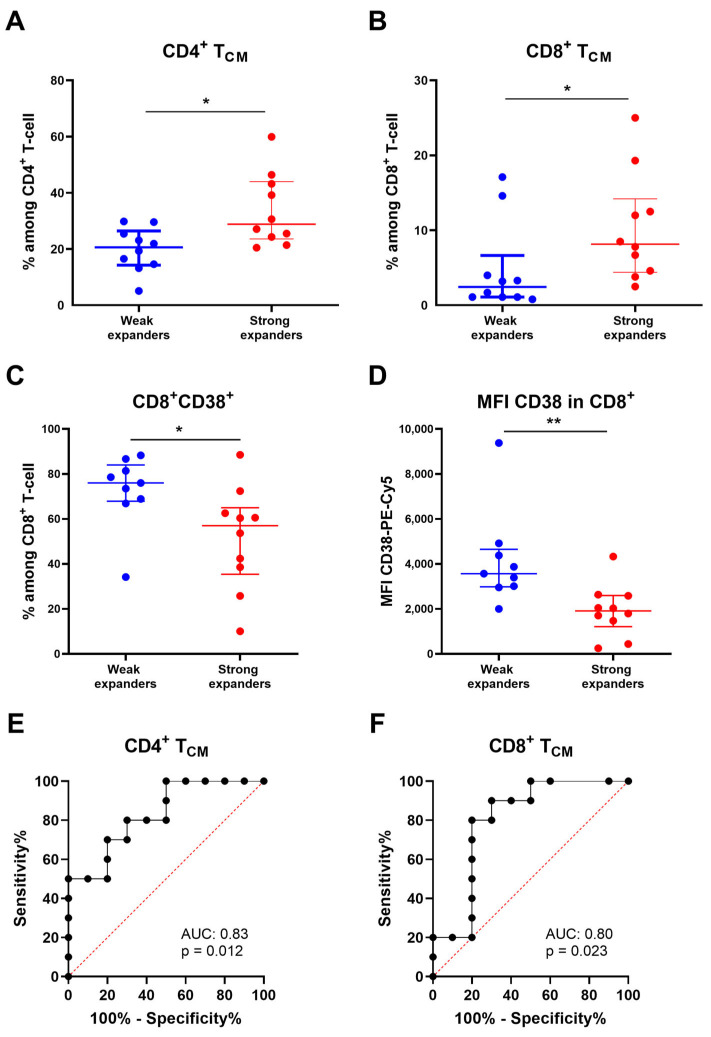
Comparison of apheresis T-cell subpopulations between weak and strong in vivo CAR-T expanders. (**A**) CD4^+^ T_CM_ cell subset comparing weak and strong in vivo CAR-T expanders. (**B**) CD8^+^ T_CM_ cell subset comparing weak and strong in vivo CAR-T expanders. (**C**) Expression of CD38 in CD8^+^ T-cell comparing weak and strong in vivo CAR-T expanders. (**D**) Mean fluorescence intensity (MFI) of CD38-Pe-Cy5 in CD8^+^ T-cell comparing weak and strong in vivo CAR-T expanders. (**E**) ROC (receiver operator characteristic) curve indicating the performance of apheresis CD4^+^ T_CM_ cells for classifying weak and strong CAR-T expanders. (**F**) ROC curve indicating the performance of apheresis CD8^+^ T_CM_ cells for classifying weak and strong CAR-T expanders. Data are presented as median with interquartile range. *p*-values between were calculated using unpaired Mann–Whitney U-tests: * *p* < 0.05, ** *p* < 0.01.

We performed T-cell immunophenotyping in fresh apheresis samples of 20/23 patients and compared T-cell subpopulations between weak and strong CAR-T expanders. Strong expanders exhibited higher values of CD4^+^ (*p* = 0.011) and CD8^+^ (*p* = 0.023) central memory T (T_CM_; CCR7^+^CD45RA^−^) cells (Figure 3A,B). Conversely, the percentage of CD8^+^ T-cells expressing CD38 in the apheresis samples was higher in weak expanders (*p* = 0.028) compared to the strong expanders (Figure 3C). Similarly, the mean fluorescence intensity (MFI) of CD38 antigen on CD8^+^ T-cells was higher in the weak expander group (*p* = 0.003) (Figure 3D). No significant differences were observed in other T-cell subsets, including regulatory, senescent or in CD4/CD8 expression between the two groups (Table 2). To evaluate the discriminative ability of apheresis T_CM_ cells with respect to weak/strong CAR-T expansions, we plotted receiver operating characteristic (ROC) curves. Among CD4^+^ T_CM_ cells, the area under the curve (AUC) was 0.83 (95% CI: 0.65–1.00; *p* = 0.012), and a cut-off value of 25.5% was proposed with a sensitivity of 70% (95% CI: 39.68–89.22%) and a specificity of 80% (95% CI: 49.02–96.45%) (Figure 3E). In addition, we obtained an AUC of 0.80 (95% CI: 0.58–1.00; *p* = 0.023) for CD8^+^ T_CM_ cells, and a cut-off value of 4.3% was proposed with a sensitivity of 80% (95% CI: 49.02–96.45%) and a specificity of 80% (95% CI: 49.02–96.45%) (Figure 3F).

### 2.4. Apheresis T-Cell Subsets Correlate with Clinical Outcomes

**Figure 4:**
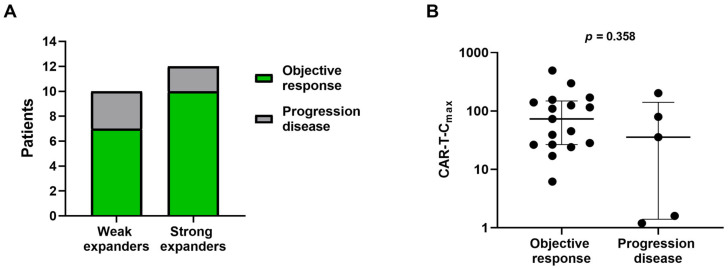
Association between CAR T-cell expansion and clinical efficacy three months after treatment. (**A**) ORRs comparing weak and strong in vivo CAR-T expanders. (**B**) Association of CAR-T C_max_ with treatment response. Patients classified as objective response include those achieving complete or partial response. Data are presented as median with interquartile range.

Correlation analyses were conducted to assess the association between CAR T-cell expansion, apheresis T-cell subsets and treatment efficacy. Clinical response was assessed by positron emission tomography with computed tomography (PET-CT) in 22 of the 23 patients, with a median follow-up of 10 months (IQR: 3–18 months) after CAR T-cell infusion. At three months, the overall response rate (ORR) for the entire cohort was 77.3%, with ORRs of 83.3% for strong expanders and 70% for weak expanders (Figure 4A). No significant differences in CAR-T-C_max_ values were observed between responders and non-responders at three months (Figure 4B).

**Figure 5:**
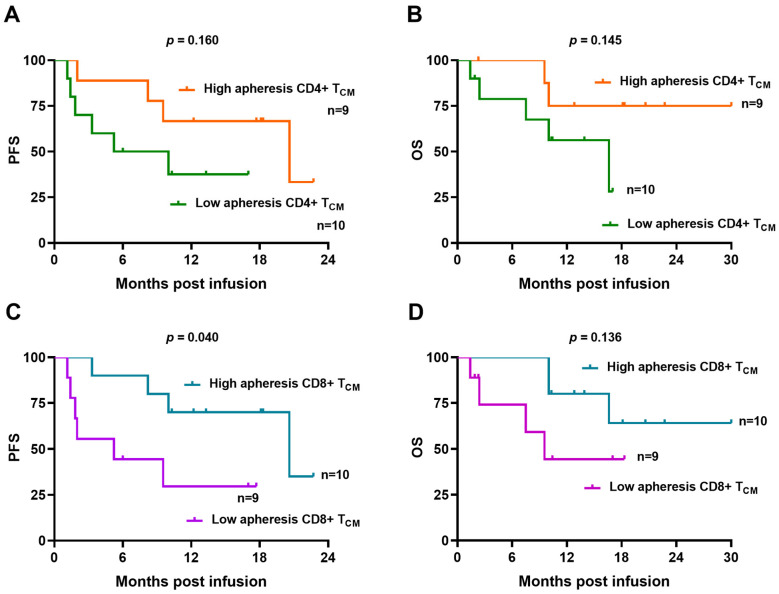
Immunophenotypic characteristics of apheresis T-cell subsets correlate with clinical outcome. (**A**,**B**) Kaplan–Meier curves of patients with high (>25.5%) and low (<25.5%) apheresis CD4+ TCM cell subset showing PFS and OS. (**C**,**D**) Kaplan–Meier curves of patients with high (>4.3%) and low (<4.3%) apheresis CD8^+^ T_CM_ cell subset showing PFS and OS. Comparisons are made applying the log-rank test.

We also performed a comparison of apheresis T-cell subsets between responders and no responders at 3 months, with no significant differences observed (Table S1). Progression-free survival (PFS) and overall survival (OS) were compared between strong and weak expanders, with similar outcomes observed (Figure S5A,B). We also explored the potential of central memory T-cell signature in apheresis samples on CAR T-cell efficacy. Patients were stratified based on the proposed cut-off values for CD4^+^ T_CM_ (25.5%) and CD8^+^ T_CM_ (4.3%) cells determined from apheresis samples. Patients with high values of CD4^+^ T_CM_ cells exhibited a trend toward improved PFS and OS curves, although the differences did not reach statistical significance (Figure 5A,B). However, patients with higher values of CD8^+^ T_CM_ cells in apheresis samples exhibited significantly longer PFS at 12-month follow-up compared with those exhibiting lower values (70.0 vs. 29.6, respectively, *p* = 0.04) (Figure 5C). In addition, OS at 12 months was 80.0% for patients with higher values of apheresis CD8^+^ T_CM_ compared with 44.4% for those with lower values, although the difference was not statistically significant (Figure 5D).

## 3. Discussion

Despite the favourable outcomes achieved with axi-cel in r/r LBCL, a proportion of patients still fail to respond or eventually relapse [3,8]. Identifying biomarkers is essential to optimize patient selection and treatment efficacy, particularly given the prolonged manufacturing process of commercial CAR T-cells, the potential risk of toxicities and the availability of newly approved immunotherapies [14,15]. Making timely therapeutic decisions is crucial to ensure optimal treatment outcomes. Therefore, we aimed to assess the association between T-cell phenotypes in the initial apheresis with CAR T-cell kinetics and clinical outcome.

We evaluated a cohort of 23 patients with LBCL who received axi-cel treatment in a real-world clinical setting. The success of CAR T-cell therapy relies on the ability of the transferred cells to proliferate, effectively trafficking to tumour sites, persist over time and execute cytotoxic effector functions [16]. We detected circulating CAR T-cells in all patients, with a CAR-T-C_max_ occurring predominantly at day 7 after infusion, consistent with findings reported in pivotal trial [3]. We defined patients as weak or strong CAR-T expanders based on the median CAR-T-C_max_ and evaluated the putative correlation between T-cell subsets in their initial apheresis.

As an autologous therapy, compromised apheresis-derived T-cells lead to CAR products with different memory phenotype, functional activation and exhaustion status. To date, few studies have examined apheresis products, mainly in the context of tisagenlecleucel therapy, demonstrating that higher proportions of naïve/stem-cell-like T-cell subsets are associated with enhanced CAR T-cell expansion, persistence and antitumour activity [17,18,19], whereas another study has reported that a higher proportion of effector CD3^+^CD27^−^CD28^−^ T-cells correlates with inferior outcomes [20]. However, this has not been extensively investigated in axi-cel therapy, where one study reported that naïve T-cell phenotypes in both the apheresis and the final CAR T-cell product were associated with enhanced response durability [21]. We analysed lymphocyte subpopulations in apheresis products, stratified into CD4^+^ and CD8^+^ compartments, and no significant correlation was observed between CD4 or CD8 naïve T-cell and CAR-T response. Frequencies of naïve cells were reduced in our cohort, likely due to an older median age compared to the aforementioned study, which could have masked potential correlations. Interestingly, we observed that the quantity of CD8^+^ T_CM_ cells in apheresis products positively impacts on clinical outcomes following axi-cel therapy. Patients with higher frequencies of CD8^+^ T_CM_ cells in the apheresis product exhibited significantly improved PFS at 12 months, and a trend toward better OS was noted, although it did not reach statistical significance. This tendency was also observed when patients were stratified according to the quantity of CD4^+^ T_CM_ cells in the apheresis product, although the differences were not statistically significant. Our findings suggest that CD8^+^ T_CM_ cells may contribute to treatment efficacy; however, this association for CD4^+^ T_CM_ cells requires further investigation. This observation emphasizes the importance of separately evaluating CD4^+^ and CD8^+^ subsets to better identify T-cell populations associated with clinical outcomes and to reduce the heterogeneity inherent to analyses of total T-cells.

We further confirmed that strong axi-cel expansions are associated with a higher percentage of CD4^+^ T_CM_ and CD8^+^ T_CM_ cells in the initial apheresis. In line with this, previous reports identified that CAR CD8^+^ T-cells with a central memory phenotype are associated with improved expansion and response [13,22]. Functionally, central memory T-cells are experienced cells capable of producing IL-2 and effector cytokines upon stimulation and exhibit lymphoid homing profiles and high proliferative capacity [23]. In addition, a positive correlation between T-cell phenotypes in the apheresis material and in the final CAR product has also been reported [21]. Therefore, although we were not able to analyse the final product, our approach indicates feasibility and highlights the potential to identify cellular biomarkers before the expensive manufacturing process. In addition, our exploratory study may contribute to the development of optimized production strategies, including approaches aimed at enriching clinically relevant cell populations.

Our data contribute to identifying the impact of apheresis T_CM_ phenotypes over CAR T-cell kinetics and to demonstrating a correlation between the apheresis CD8^+^ T_CM_ cells and axi-cel outcome. In addition, we propose a cut-off point for CD8^+^ T_CM_ cells as a pre-treatment blood biomarker of response to axi-cel. Interestingly, we also observed a negative correlation between the percentage of CD8^+^CD38^+^ T-cells in apheresis and the CAR-T expansion. CD38 antigen was initially identified as a marker of T-cell activation [24], although its expression has also been associated with T-cell exhaustion in chronic proinflammatory contexts [25]. In agreement with our results, a recent study reported a positive association between CD38 expression and CAR T-cell exhaustion [26].

No correlation was observed between CAR T-cell expansion and clinical response, consistent with heterogeneous results previously reported in r/r lymphoma [3,4,13,27,28,29]. The lack of correlation may reflect the influence of additional biological and clinical factors beyond early in vivo expansion, including tumour microenvironment-mediated immunosuppression, inter-patient heterogeneity in disease burden and prior therapies. Importantly, CAR T-cell expansion alone does not necessarily predict clinical efficacy; rather, the persistence and functional quality of the infused cells are critical determinants of long-term therapeutic outcomes. Notably, three patients categorized as weak expanders have remained in clinical remission for 18 months or longer. Several factors have been identified as contributing to durable responses following CAR-T therapy [30]. Collectively, our results suggest that long-term outcomes may also depend on a memory subset of CAR T-cells. On the other hand, CAR T-cell expansion has been clearly associated with CRS and ICANS [31,32,33]. However, we did not observe correlation between CAR-T-C_max_ and toxicities, consistent with findings from other lymphoma studies [28,29]. This may be due to improved patient management and early administration of immunomodulatory therapies.

Our study has some limitations to consider. The findings are restricted to axi-cel and may not generalize to other CAR-T therapies. Due to the small sample size and limited number of events, we were unable to fully assess the independent impact of clinical factors such as tumour burden and prior therapies on post-CAR-T outcomes. Analysis of the final CAR-T products was not possible, limiting correlation assessments. Further validation in independent cohorts is needed, and proposed cut-off values should be interpreted with caution. Nevertheless, our results highlight the potential relevance of characterizing patient pre-treatment T-cells for improved selection of CAR-T therapy candidates and prognostic assessment.

## 4. Materials and Methods

### 4.1. Study Cohort

This study presents data from 23 patients with r/r LBCL who received axi-cel, a CD19-targeted CAR T-cell therapy, between December 2022 to March 2025 at Ramon y Cajal University Hospital. All patients underwent lymphodepletion according to established protocol [3]. Clinical responses were assessed by PET-CT at month 1 and at month 3, or when clinically indicated. CRS and ICANS were defined and graded according to American Society for Transplantation and Cellular Therapy guidelines [34].

### 4.2. Samples

CAR T-cell monitoring was carried out by multiparametric flow cytometry on heparin anticoagulated peripheral blood as a part of routine follow-up. An additional flow cytometry assay was performed on fresh apheresis samples to characterize cell immunophenotypes.

### 4.3. Monoclonal Antibodies

For the monitoring of commercial CD19 CAR T-cells post-infusion, the monoclonal antibodies used in the analysis of peripheral blood included CD45-V500, CD3-APC, CD4-PE, CD8-APC-H7, CD33-PE-Cy5 and CD56-PE-Cy7, all from BD-Biosciences. As a CAR detection reagent, we used an FITC-labelled CD19-recombinant human protein (Acrobiosystems, Nework, DE, USA). A fluorescence minus one (FMO) control tube was included to accurately determine the cut-off for CAR T-cell positivity. Panel design and gating strategies were defined according to published recommendations for multiparametric flow cytometry methods in chimeric antigen receptor T-cell analyses [35,36].

For immunophenotypic characterization of fresh apheresis samples, cells were labelled with the following set of antibodies: CD45-V500, CD45RA-FITC, CCR7-PE, CD3-PerCP, CD3-BV421, CD25-PE-Cy7, CD4-APC, CD4-PE-Cy7, CD8-APC-H7, CD127-BV421, CD57-FITC, CD28-PE-Cy5 and CD38 PE-Cy5, all from BD-Biosciences. The antibody panel was selected to enable the phenotypic characterization of distinct T-cell subpopulations, based on established differentiation and functional markers described in the literature [37]. Dual CD28/CD57 staining was used to evaluate T-cell senescence state, as previously described [38].

### 4.4. Labelling of Surface Molecules

Peripheral blood samples were surface-stained for 20 min at room temperature in the dark with adequate amounts of fluorescence-labelled monoclonal antibodies. After incubation, red blood cells were lysed using lysing solution (BD Biosciences, San Jose, CA, USA) following mixing and 10 min incubation in the dark. Lysed cells were centrifuged at 2000 rpm for 5 min and washed 3 times with phosphate-buffered saline (PBS). Finally, stained cells were resuspended in FACS solution and analysed by flow cytometry as detailed below. Fresh apheresis samples were surface-stained for 20 min at room temperature in the dark with adequate amounts of fluorescence-labelled monoclonal antibodies. After incubation, red blood cells were lysed using ammonium chloride 1× (BD Biosciences) following mixing and 10 min incubation in the dark. Lysed cells were centrifuged at 2000 rpm for 5 min and washed once with PBS. Finally, stained cells were resuspended in FACS solution and analysed by flow cytometry as detailed below.

### 4.5. Flow Cytometry

Follow-up blood samples were processed within 24 h of collection, and apheresis samples within 1–2 h. A minimum of 1 × 10^5^ events in peripheral blood samples or fresh apheresis were analysed within 1 h after antigen labelling using a FACSCanto II (BD, Biosciences). Isotype controls were used to set the mean autofluorescence values. The percentages of every cell subset over total analysed cells were obtained by the FACSDiva software V.8.0 (BD Biosciences, San Jose, CA, USA). The gating strategies for CAR T-cell detection are shown in Figure S1, and those for the apheresis cell characterization are shown in Figures S2 and S3.

### 4.6. Statistical Analysis

Statistical analyses were performed with GraphPad Prism 8.0 software (GraphPad Prism Inc., San Diego, CA, USA). Differences in cell subpopulations between weak and strong CAR-T expanders were assessed by two-sided Mann–Whitney U-tests. PFS and OS were evaluated using Kaplan–Meier survival curves and compared with the log-rank test. PFS was defined as the time from CAR-T infusion to progression, relapse or death by any cause. OS was defined as the time from CAR-T infusion to death by any cause. We calculated the area under the receiver operating characteristic (ROC) curve to evaluate the accuracy of apheresis T-cell subpopulations to predict axi-cel kinetics and clinical outcome. Optimal cut-off values were determined based on the maximum Youden index, balancing sensitivity and specificity. *p* values below 0.05 were considered significant.

## 5. Conclusions

To summarize, our study identifies that a high quantity of CD8^+^ T_CM_ cells in apheresis samples is associated with strong CAR-T expansion and improved clinical outcome in r/r LBCL undergoing axi-cel therapy. This novel biomarker has the potential to improve the selection of patients for axi-cel therapy. In addition, a high quantity of CD4^+^ T_CM_ cells in apheresis samples was also associated with strong CAR-T expansion. Conversely, we observed that elevated proportions of CD8^+^CD38^+^ T-cells in apheresis samples constitute a biomarker associated with weaker CAR-T expansions. Taken together, our findings underscore the value of identifying cellular biomarkers prior to the resource-intensive CAR-T manufacturing process. Early stratification based on these parameters could inform therapeutic decision-making and guide the development of strategies aimed at improving efficacy rates. Importantly, this approach represents a feasible and scalable strategy for integration into routine clinical practice, as the biomarker can be easily incorporated into existing workflows. Nevertheless, further validation in independent cohorts is required to confirm its potential to enhance patient selection and optimize clinical outcomes.

## Figures and Tables

**Table 1:** Clinical characteristics of the patients included in the study.

	N (%)/Median (IQR)
Age, years	65 (54.5–70)
Sex	
Male	14 (61%)
Female	9 (39%)
Lymphoma subtype	
LBCL	22 (96%)
DLBCL NOS	16 (70%)
HGBCL *	4 (17%)
PTLD DLBCL NOS	1 (4%)
PMBCL	1 (4%)
FL	1 (4%)
Histological transformation from FL	LBCL
No	15 (68%)
Yes	7 (32%)
Prior lines of treatment	
1	3 (13%)
2	18 (78%)
3–5	2 (9%)
Autologous HSCT	5 (21.7%)
CRS	23 (100%)
Grade 0	0
Grade 1	5 (22%)
Grade 2	17 (74%)
Grade 3	1 (4%)
Grade 4	0
ICANS	12 (51%)
Grade 0	11 (48%)
Grade 1	7 (30%)
Grade 2	1 (4%)
Grade 3	3 (13%)
Grade 4	1 (4%)
Treatment response month + 3	
Objective response	17 (77.3%)
Progression disease	5 (22.7%)
No information	1

LBCL—large B-cell lymphoma; DLBCL NOS—diffuse large B-cell lymphoma, not otherwise specified; HGBCL—high-grade B-cell lymphoma; PTLD—post-transplant lymphoproliferative disorder; PMBCL—primary mediastinal B-cell lymphoma; FL—follicular lymphoma; HSCT—hematopoietic stem cell transplantation; CRS—cytokine release syndrome; ICANS—immune effector cell-associated neurotoxicity syndrome. * Three-quarters with MYC, BCL2 and BCL6 rearrangements; one-quarter with MYC and BCL2 rearrangements.

**Table 2:** Comparison of apheresis T-cell subpopulations between weak and strong in vivo CAR-T expanders.

	Percentages [Subset]		
	Weak Expanders	Strong Expanders	*p*
T-lymphocytes [CD45]	39.2 (19.8–56.6)	53.9 (42.5–59.7)	ns
CD4^+^CD8^−^ [CD3]	43.7 (31.9–64.9)	45.8 (29.1–61.6)	ns
CD4^−^CD8^+^ [CD3]	48.5 (25.7–57.9)	40.3 (30.5–66.1)	ns
CD4^+^CD8^+^ [CD3]	1.0 (0.6–2.6)	1.5 (1.0–2.6)	ns
CD4^−^CD8^−^ [CD3]	1.5 (1.2–6.4)	2.8 (2.0–6.5)	ns
Ratio CD4/CD8 [CD3]	0.9 (0.5–2.5)	1.1 (0.4–2.0)	ns
CD4			
Treg [CD4]	8.8 (7.2–12.8)	10.6 (5.8–13.4)	ns
Naïve [CD4]	1.8 (0.4–23.4)	7.0 (2.5–13.4)	ns
Central memory [CD4]	20.6 (14.2–26.4)	28.9 (23.6–44.0)	0.011
Effector memory [CD4]	69.6 (47.8–82.5)	60.1 (46.2–65.8)	ns
Terminally differentiated [CD4] CD28^+^CD57^−^ [CD4] CD28^+^CD57^+^ [CD4] CD28^−^CD57^−^ [CD4] CD28^−^CD57^+^ [CD4] CD38^+^ [CD4] MFI CD38-PE-Cy5 [CD4]	1.7 (0.9–19.6) 94.6 (73.6–97.6) 1.8 (0.8–2.4) 1.4 (0.1–3.6) 2.5 (0.15–19) 51.5 (40.3–61.4) 2185 (1702–4111)	1.6 (0.7–3.0) 94.7 (68.3–99.3) 1.5 (0.4–3.6) 0.7 (0.2–5.5) 1.9 (0.2–20.9) 35.8 (26.8–49.7) 1307 (1082–1836)	ns ns ns ns ns ns 0.044
CD8			
Naïve [CD8]	2.0 (1.0–14.8)	6.3 (2.9–10.0)	ns
Central memory [CD8]	2.4 (1.1–6.6)	8.2 (4.4–14.2)	0.023
Effector memory [CD8]	47.1 (30.2–70.7)	54.3 (37.1–59.9)	ns
Terminally differentiated [CD8] CD28^+^CD57^−^ [CD8] CD28^+^CD57^+^ [CD8] CD28^−^CD57^−^ [CD8] CD28^−^CD57^+^ [CD8] CD38^+^ [CD8] MFI CD38-PE-Cy5 [CD8]	33.2 (25.2–61.8) 45.4 (35.0–52.2) 2.3 (1.0–4.2) 21.8 (9.6–31.1) 33.4 (16.0–41.0) 76.0 (67.9–84.1) 3569 (2987–4651)	25.5 (21.5–45.2) 47.6 (42.2–83.6) 1.9 (1.7–5.4) 13.3 (7.9–23.6) 24.3 (5.2–36.6) 57.1 (35.4–65.1) 1915 (1216–2600)	ns ns ns ns ns 0.028 0.003

The results of characterization of 20 fresh apheresis samples by flow cytometry are shown. T-cell subpopulations were identified as follows: naïve (CCR7^+^CD45RA^+^), central memory (CCR7^+^CD45RA^−^), effector memory (CCR7^−^CD45RA^−^), terminally differentiated (CCR7^−^CD45RA^+^) and Treg (CD4^+^CD25^+^CD127^low/−^). Percentages or mean fluorescence intensity (MFI) refer to the subset included in square brackets [ ]. Results are expressed as median (25–75% IQR). *p*-values below 0.05 are reported; values ≥ 0.05 were considered not statistically significant (ns).

## Data Availability

Due to the inclusion of sensitive patient data, the datasets generated and/or analysed during the current study are not publicly available. However, they can be made available from the corresponding author upon reasonable request, subject to ethical and legal approvals.

## Supplementary Materials

The following supporting information can be downloaded at: https://www.mdpi.com/article/10.3390/ijms27020866/s1.

## Abbreviations

The following abbreviations are used in this manuscript:

CAR	Chimeric antigen receptor
r/r	Relapsed or refractory
DLBCL	Diffuse large B-cell lymphoma
LBCL	Large B-cell lymphoma
Axi-cel	Axicabtagén ciloleucel
T_CM_	Central memory T-cells
FL	Follicular lymphoma
PMBCL	Primary mediastinal B-cell lymphoma
HSCT	Hematopoietic stem cell transplantation
CRS	Cytokine release syndrome
ICANS	Immune effector cell-associated neurotoxicity syndrome
ROC	Receiver operating characteristic
MFI	Mean fluorescence intensity
PET	Positron emission tomography
ORR	Overall response rate
PFS	Progression-free survival
OS	Overall survival
PBS	Phosphate-buffered saline
