# Supplementary material for: Apheresis CD8+CCR7+CD45RA− T-Cells as a Novel Biomarker Associated with CAR T-Cell Kinetics and Clinical Outcome
Source: Int J Mol Sci. 2026 Jan 15;27(2):866. doi: 10.3390/ijms27020866 (PMC12841578; doi:10.3390/ijms27020866)
Supplement: Supplementary file 1 [file ijms-27-00866-s001.zip › ijms-4055260-supplementary.pdf]

Supplementary Materials for:

Apheresis $CD8^+CCR7^+CD45RA^-$ T cells as a novel biomarker associated to CAR-T cell kinetics and clinical outcome

Iván García de la Torre[1], Carlota García-Hoz[1], Fernando Martin-Moro[2], José Ignacio Fernández-Velasco[1], Kyra Velázquez-Kennedy[2], Eulalia Rodríguez-Martín[1], Alejandro Luna De Abia[2], Ernesto Roldán[1], Gemma Moreno Jiménez[2], Javier López-Jiménez[2], Luisa María Villar[1], Roberto Pariente-Rodríguez[1*].

[1] Department of Immunology, Hospital Universitario Ramón y Cajal, Instituto Ramón y Cajal de Investigación Sanitaria (IRYCIS), Madrid, Spain; ivandelatorre.bio@gmail.com (I.G.T.); carlota.garcia-hoz@salud.madrid.org (C.G.-H.); jfvelasco@salud.madrid.org (J.I.F.-V.); lali_rmartin@yahoo.es (E.R.-M.); ernesto.roldan@salud.madrid.org (E.R.); luisamaria.villar@salud.madrid.org (L.M.V.); roberpariente@gmail.com (R.P.-R).

[2] Department of Hematology, Hospital Universitario Ramón y Cajal, Instituto Ramón y Cajal de Investigación Sanitaria (IRYCIS), Madrid, Spain; fmartinmoro@usal.es (F.M.-M.); kyra.velazquez@salud.madrid.org (K.V.-K); lunadeabia@gmail.com (A.L.A.) gemma.moreno@salud.madrid.org (G.M.J.); jljimenez@salud.madrid.org (J.L.-J.).

* Correspondence: roberpariente@gmail.com (R.P.-R.).

Table of Contents:

Figure S1: Immunomonitoring of in vivo CAR-T cell expansion.

Figure S2: Study of T-cells subpopulations in apheresis sample.

Figure S3: Study of activation/senescence marker expression in apheresis sample.

Figure S4: Comparison of axi-cel CD4/CD8 ratio between weak and strong expanders at the time of CAR-T-C_{max}.

Table S1: Comparison of apheresis T-cell subpopulations between patients with objective response and progression disease after 3 months of axi-cel treatment.

Figure S5: Comparison of long-term outcome between weak and strong expanders.

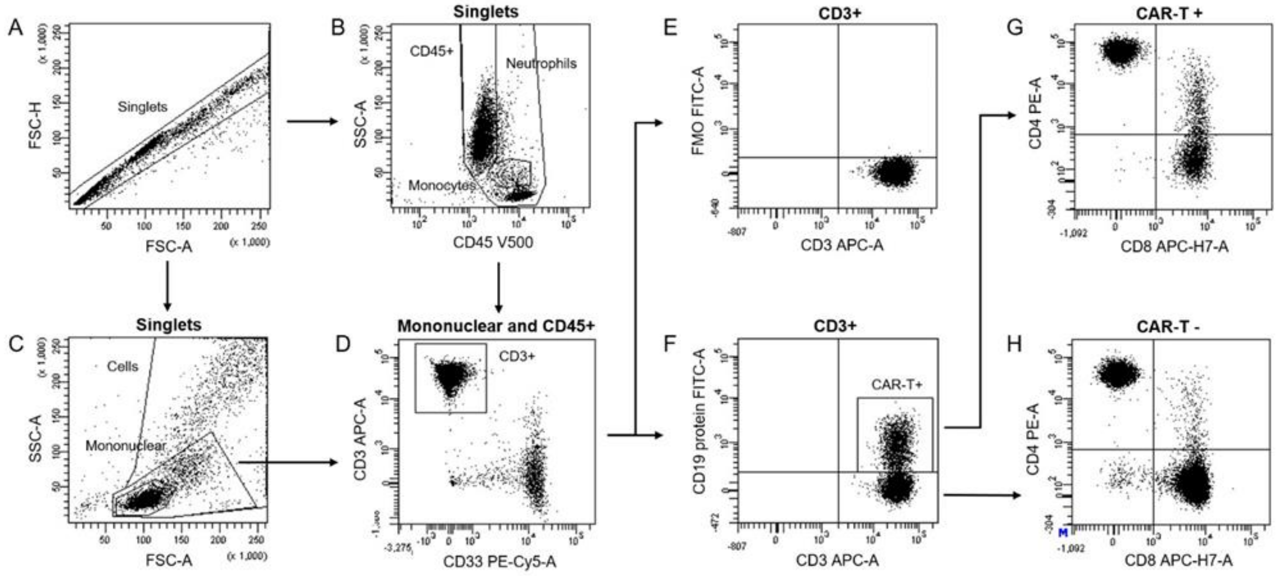

Figure S1. Immunomonitoring of *in vivo* CAR-T cell expansion. **(A)** Doublets were excluded from the analysis. **(B)** CD45$^+$ cells were gated. **(C)** Selection of viable cells based on forward scatter (FSC) and side scatter (SSC) characteristics. Mononuclear cells were gated by FSC and SSC. **(D)** T lymphocytes were identified from mononuclear and CD45$^+$ gates, by selecting the CD3$^+$ CD33$^-$ population. **(E; F)** Using a FITC FMO control, the cutoff for positivity was established to define the CAR-T positive population. CD4 and CD8 subpopulations were analyzed both within the CAR-T positive **(G)** and the CAR-T negative fractions **(H)**.

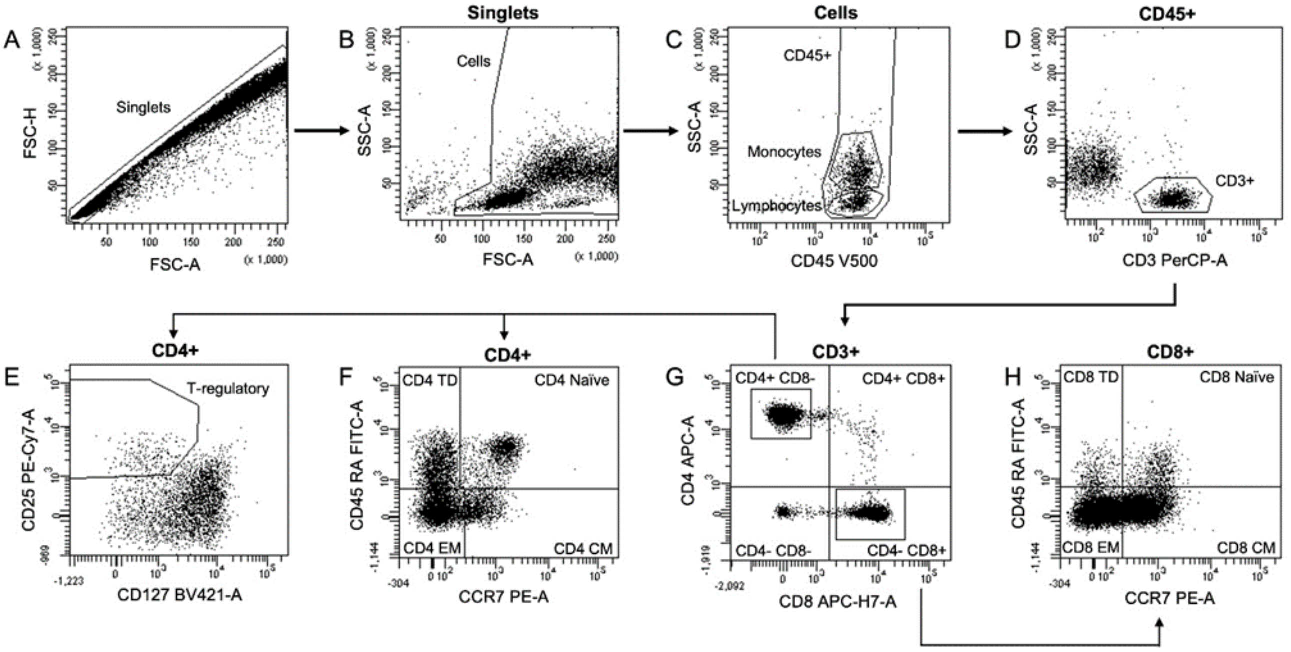

Figure S2. Study of T-cells subpopulations in apheresis sample. **(A)** Doublets were excluded from the analysis. **(B)** Selection of viable cells based on forward scatter (FSC) and side scatter (SSC) characteristics. **(C)** CD45$^+$ cells were gated. **(D)** T-cells were identified as CD45$^+$ and CD3$^+$ cells. **(G)** T helper cells were defined as CD3$^+$CD4$^+$ CD8$^-$ cells and T cytotoxic cells were defined as CD3$^+$CD4$^-$CD8$^+$ cells. **(F; H)** CD4 or CD8 subpopulations were categorised as naïve (CCR7$^+$ CD45RA$^+$), central memory (CM; CCR7$^+$CD45RA$^-$), effector memory (EM; CCR7$^-$ CD45RA$^-$), and terminally diferentiated (TD; CCR7$^-$CD45RA$^+$). **(E)** Regulatory T-cells were defined as CD3$^+$CD4$^+$ CD25^{high}CD127$^{-/low}$ cells.

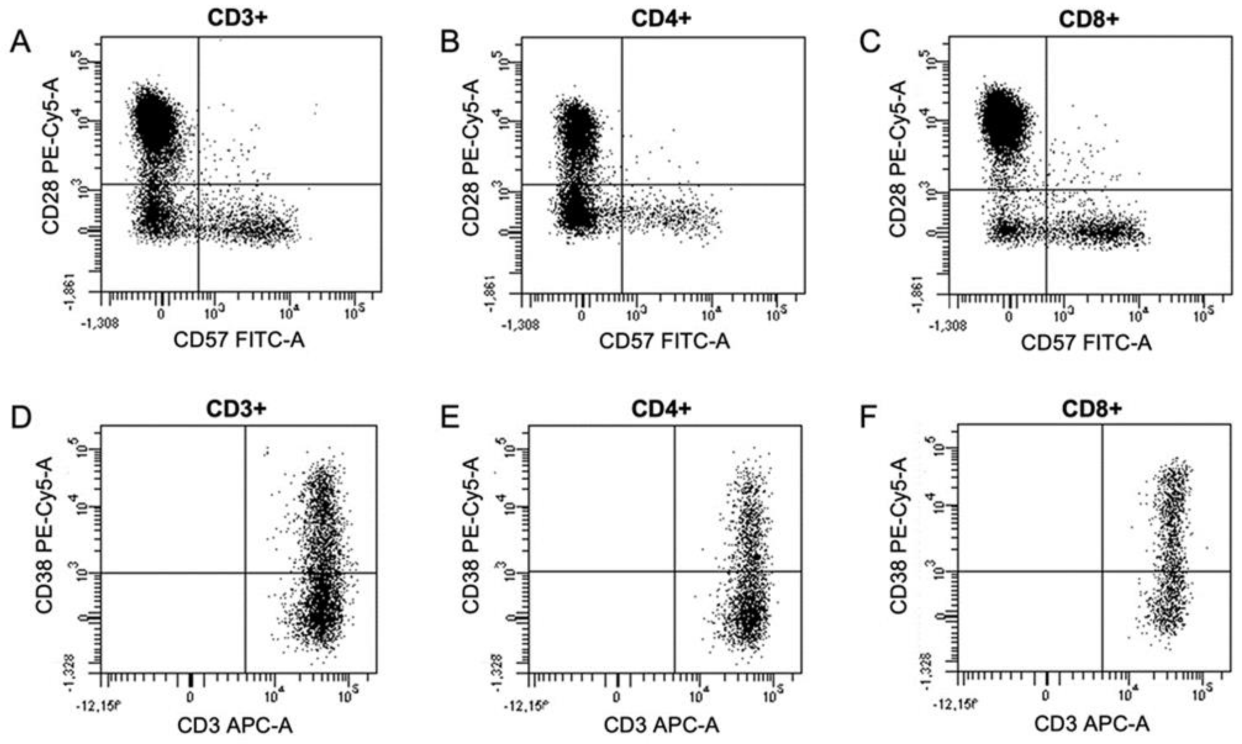

Figure S3. Study of activation/senescence marker expression in apheresis sample. CD28 and CD57 expression were analyzed in T cells **(A)**, T helper cells **(B)**, and cytotoxic T cells **(C)**. CD38 expression was analyzed in T cells **(D)**, T helper cells **(E)**, and cytotoxic T cells **(F)**.

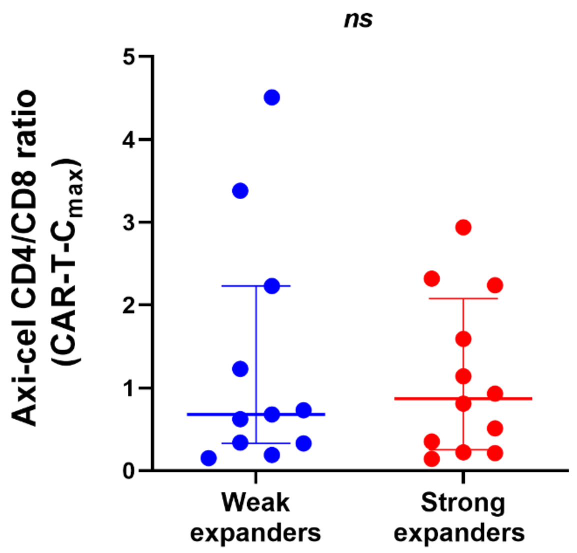

Figure S4. Comparison of axi-cel CD4/CD8 ratio between weak and strong expanders at the time of CAR-T-C_{max}. Data are presented as median with interquartile range.

Table S1. Comparison of apheresis T-cell subpopulations between patients with objective response and progression disease after 3 months of axi-cel treatment.

	Percentages [Subset]		
	Progression disease	Objective response	*p*
$CD4^+CD8^-$ [CD3]	55.0 (24.2-61.4)	41.1 (31.5-65.2)	*ns*
$CD4^-CD8^+$ [CD3]	40.1 (29.0-71.6)	50.4 (26.8-60.6)	*ns*
$CD4^+CD8^+$ [CD3]	3.3 (1.6-3.8)	1.1 (0.6-2.5)	*ns*
$CD4^-CD8^-$ [CD3]	2.2 (1.7-5.2)	2.3 (1.2-6.7)	*ns*
Ratio CD4/CD8 [CD3]	1.4 (0.3-2.1)	0.9 (0.5-2.4)	*ns*
CD4			
Treg [CD4]	13.2 (9.4-15.2)	9.1 (6.3-11.8)	*ns*
Naïve [CD4]	7.4 (1.4-17.4)	3.8 (0.5-8.6)	*ns*
Central Memory [CD4]	19.3 (15.6-24.9)	28.4 (21.2-40.2)	*ns*
Effector Memory [CD4]	63.0 (55.2-73.8)	62.5 (46.4-75.9)	*ns*
Terminally differentiated [CD4]	4.0 (1.6-13.4)	1.1 (0.7-2.6)	*ns*
$CD28^+CD57^-$ [CD4]	95.8 (76.5-97.1)	93.2 (68.4-99.3)	*ns*
$CD28^+CD57^+$ [CD4]	2.0 (1.3-3.1)	1.7 (0.5-2.7)	*ns*
$CD28^-CD57^-$ [CD4]	0.5 (0.2-2.2)	2.4 (0.1-4.8)	*ns*
$CD28^-CD57^+$ [CD4]	2.1 (0.8-18.9)	3.3 (0.2-20.9)	*ns*
$CD38^+$ [CD4]	57.0 (28.2-70.3)	41.2 (29.0-54.8)	*ns*
*MFI CD38-PE-Cy5 [CD4]	2733 (976-5384)	1525 (1097-2072)	*ns*
CD8			
Naïve [CD8]	5.5 (1.8-17.5)	3.6 (1.4-9.8)	*ns*
Central Memory [CD8]	2.5 (1.4-4.3)	8.2 (3.3-15.2)	*ns*
Effector Memory [CD8]	47.0 (32.1-57.4)	54.3 (41.5-70.7)	*ns*
Terminally differentiated [CD8]	40.0 (26.0-62.1)	25.3 (21.5-44.0)	*ns*
$CD28^+CD57^-$ [CD8]	47.2 (37.4-61.9)	47.4 (41.9-76.2)	*ns*
$CD28^+CD57^+$ [CD8]	3.4 (1.2-6.9)	2.0 (1.6-4.6)	*ns*
$CD28^-CD57^-$ [CD8]	14.1 (9.9-20.6)	17.8 (7.9-27.5)	*ns*
$CD28^-CD57^+$ [CD8]	35.4 (16.1-47.1)	24.3 (8.0-36.5)	*ns*
$CD38^+$ [CD8]	77.6 (48.5-88.4)	61.6 (37.5-74.1)	*ns*
*MFI CD38-PE-Cy5 [CD8]	3721 (1998-4215)	2316 (1775-3110)	*ns*

The results of 19 fresh apheresis characterization by flow cytometry are shown. T-cell subpopulations were identified as follows: naïve ($CCR7^+CD45RA^+$), central memory ($CCR7^+CD45RA^-$), effector memory ($CCR7^-CD45RA^-$), terminally differenciated ($CCR7^-CD45RA^+$) and Treg ($CD4^+CD25^+CD127^{low/-}$). Percentages or mean fluorescence intensity (MFI) are referred to the subset included into square brackets [ ]. Results are expressed as Median (25-75% IQR). P-values below 0.05 are reported; values ≥ 0.05 were considered not statistically significant (ns).

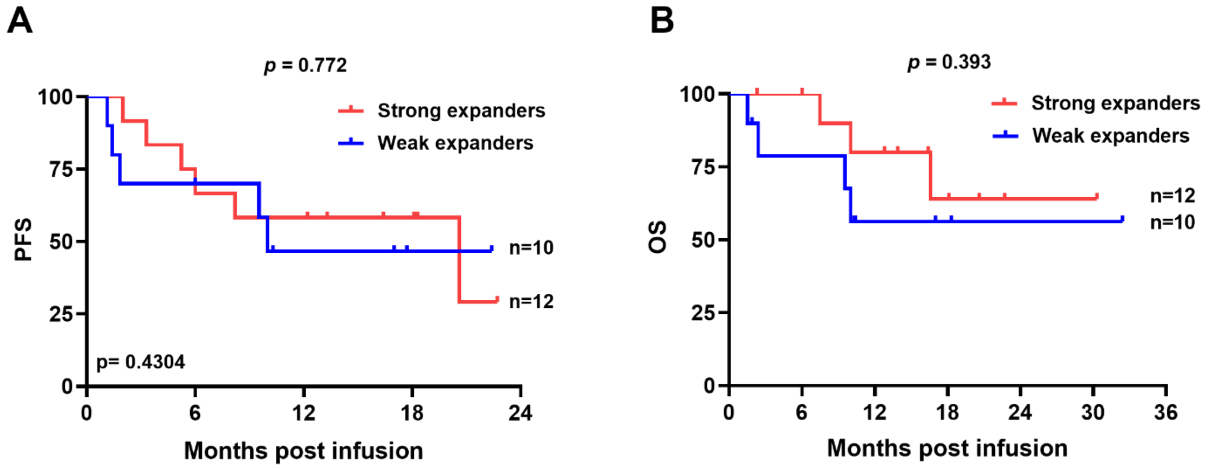

Figure S5. Comparison of long-term outcome between weak and strong expanders. Kaplan-Meier curves of weak and strong in vivo CAR-T expanders showing: **(A)** progression free-survival (PFS) and **(B)** overall survival (OS) during follow-up. Comparison are made applying the log-rank test.
